# Giant duodenal ulcers after neurosurgery for brainstem tumors that required reoperation for gastric disconnection: a report of two cases

**DOI:** 10.1186/s12893-016-0189-3

**Published:** 2016-11-17

**Authors:** Chihoko Nobori, Kenjiro Kimura, Go Ohira, Ryosuke Amano, Sadaaki Yamazoe, Hiroaki Tanaka, Kentaro Naito, Toshihiro Takami, Kosei Hirakawa, Masaichi Ohira

**Affiliations:** 1 Department of Surgical Oncology, Osaka City University Graduate School of Medicine, 1-4-3 Asahi-machi, Abeno-ku, Osaka, Japan; 2 Department of Neurosurgery, Osaka City University Graduate School of Medicine, 1-4-3 Asahi-machi, Abeno-ku, Osaka, Japan

**Keywords:** Giant duodenal ulcer, Gastric disconnection, Brainstem tumor

## Abstract

**Background:**

Despite the efficacy of pharmacotherapy for gastrointestinal ulcers, severe cases of bleeding or perforation due to gastrointestinal ulcers still occur. Giant duodenal ulcer perforation is an uncommon but difficult-to-manage pathology with a high mortality rate. We report two cases of giant duodenal ulcer perforation after neurosurgery for brainstem tumors that needed reoperation for gastric disconnection because of postoperative leakage and bleeding.

**Case presentation:**

Both cases had undergone neurosurgery for brainstem tumors, and the patients were in a shock state for several days with peritonitis due to giant duodenal perforation. In Case 1, antrectomy with Billroth II reconstruction was performed. However, reoperation for gastric disconnection was needed because of major leakage of gastrojejunostomy and jejunojejunostomy. In Case 2, an omental patch, cholecystectomy, and insertion of a bile drainage tube from the cystic duct were performed for the giant duodenal ulcer, but leakage and bleeding from the ulcer edge required reoperation for gastric disconnection.

**Conclusions:**

Brainstem tumors in these cases might have been related to duodenal ulcer perforation with late diagnosis that progressed to severe sepsis. For giant duodenal ulcer perforation with poor general condition, simple closure including omental patch or antrectomy with reconstruction is hazardous. Antrectomy with gastric disconnection, meaning gastrostomy, duodenostomy, feeding jejunostomy and cholecystectomy, is recommended.

## Background

Cushing reported gastroduodenal ulcers produced by elevated intracranial pressure caused by an intracranial tumor, head injury, or other space-occupying lesion, which have been called Cushing’s ulcer [1]. The use of histamine H2-receptor antagonists or proton pump inhibitors can decrease the incidence of Cushing’s ulcer and its complications, such as bleeding and perforation. However, cases of severe bleeding or perforation from gastroduodenal ulcers still occur. Generally, duodenal ulcer perforation is a surgical emergency. Factors such as advanced age, concomitant disease, preoperative shock, large size of the perforation, and delays in presentation and operation have been identified as risk factors for mortality from duodenal ulcer perforation [2]. Gapta et al. classified duodenal ulcer perforations into three groups based on the size of the perforations: ‘small’ perforations less than 1 cm in diameter; ‘large’ perforations more than 1 cm but less than 3 cm in diameter; and ‘giant’ perforations exceeding 3 cm [2]. Small and large perforations are common and relatively easy to manage, resulting in low mortality rates. On the other hand, giant perforations are uncommon but difficult to manage and associated with higher mortality rates. Simple closure or omental patching alone have been reported as unsafe. Two cases of giant duodenal ulcer perforation after neurosurgery that needed re-operation because of postoperative leakage and bleeding are described. Taking these cases into account, we discuss how to cope with perforation of a giant duodenal ulcer that has progressed to sepsis because of late diagnosis.

## Case presentations

**Fig. 1:**
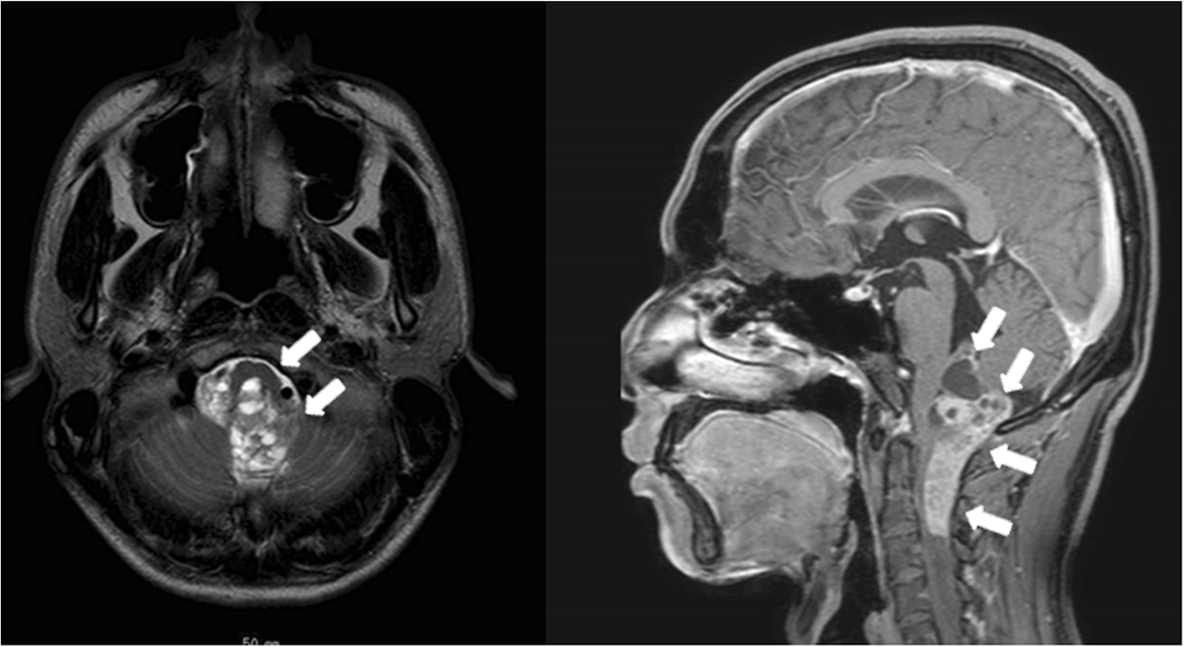
Head MRI. The MRI scan reveals an anaplastic ependymoma that extended from brainstem to forth ventricle

Case 1 involved a 25-year-old man who had undergone surgical resection of anaplastic ependymoma extending from the brainstem to the fourth ventricle (Fig. 1). Two days after neurosurgery, laboratory data showed an unexpectedly severe inflammatory response (white cell count, 18,900/μL; C-reactive protein (CRP), 12.8 mg/dl). The patient was observed with administration of meropenem.

**Fig. 2:**
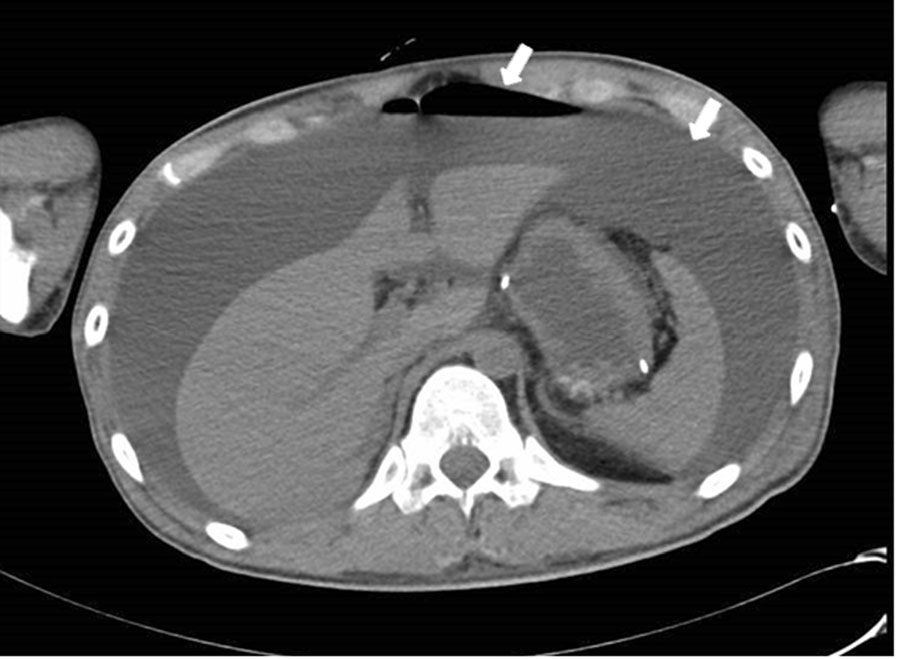
Abdominal CT. The CT scan reveals a considerable amount of fluid and free air (arrow)

**Fig. 3:**
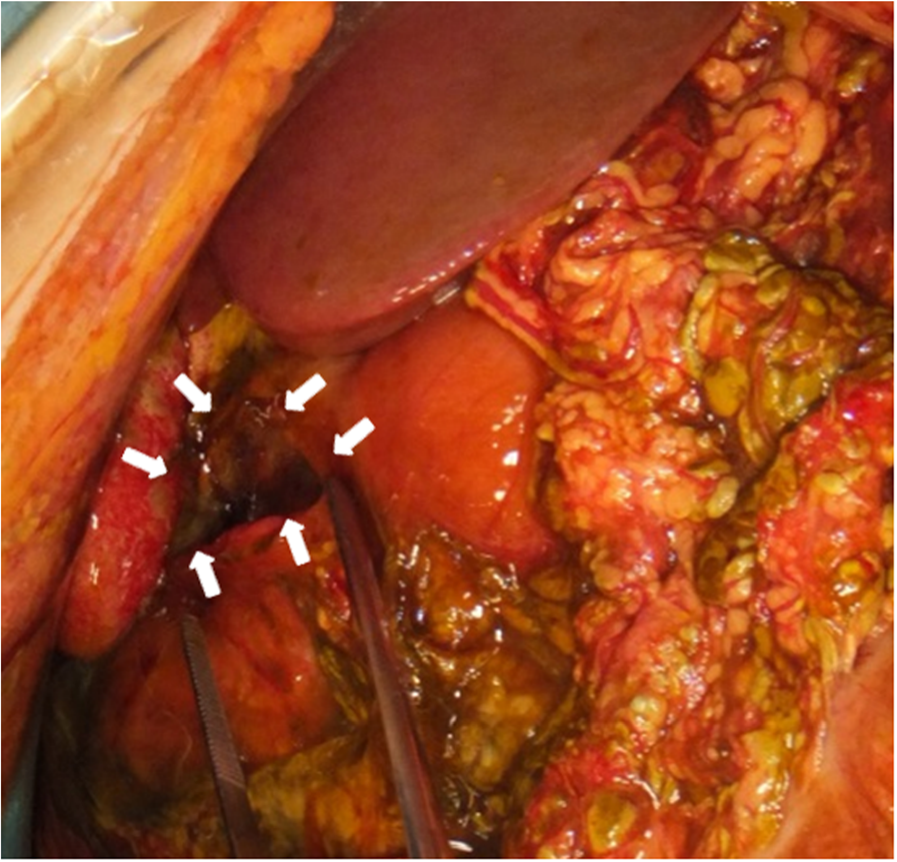
The intra-operative finding. The perforation 3.5-cm in diameter was found in the second portion of the duodenal bulb

Two days later, he developed shock and the abdomen appeared severely distended. Vital signs were: temperature, 39.1 °C; heart rate, 130 beats/min; blood pressure, 73/37 mmHg under medication with dopamine 8 μg/kg/min and noradrenaline 0.25 μg/kg/min; and oxygen saturation, 94 % in room air. Laboratory data showed: white cell count, 23,100/μL; platelet count, 32,000/μL; CRP, 5.48 mg/dL. Computed tomography (CT) showed free air and massive ascites (Fig. 2), and emergency surgery was performed under a presumptive diagnosis of gastrointestinal perforation. On laparotomy, 3 L of muddy ascites was removed, and a perforation 3.5 cm in diameter was found in the second portion of the duodenal bulb (Fig. 3). Antrectomy including the ulcerated portion using Billroth II reconstruction with Braun anastomosis, insertion of a duodenal drainage tube from the duodenal stump, and cholecystectomy with insertion of a bile drainage tube from the cystic duct were performed.

Ten days after the ulcer operation, major leakage of the gastrojejunostomy and jejunojejunostomy required re-operation, involving gastric disconnection, gastrostomy, duodenostomy, and feeding jejunostomy. After reoperation, the patient developed multiple-organ failure, but he recovered with intensive care. Eight months after the reoperation, digestive tract reconstruction surgery was performed using the Roux-en-Y method. Since that reconstruction surgery, the patient has been making satisfactory progress.

**Fig. 4:**
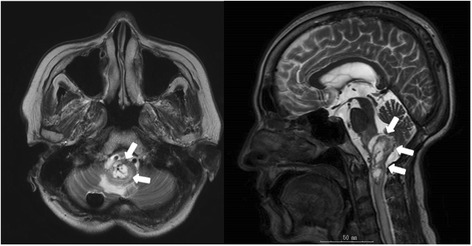
Head MRI. The MRI scan reveals an a brainstem hemangioblastoma

**Fig. 5:**
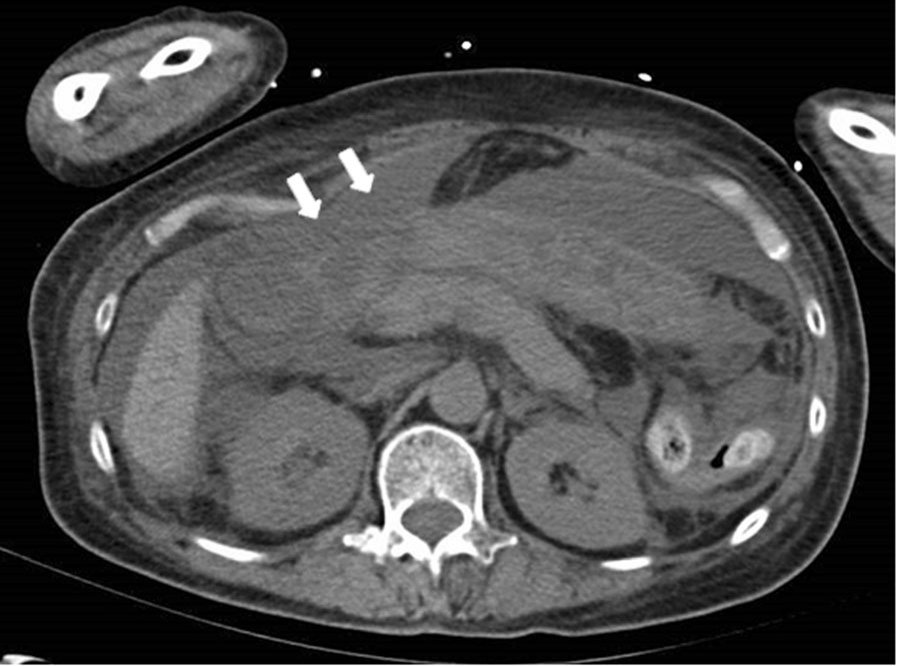
Abdominal CT. The CT scan reveals massive fluid accumulation and an irregular duodenal wall

Case 2 involved a 62-year-old woman. She had undergone surgical resection of a brainstem hemangioblastoma that progressed acutely after stereotactic radiosurgery (Fig. 4). Six days after neurosurgery, laboratory data revealed an unexpectedly severe inflammatory response (white cell count, 23,100/μL; CRP, 18.5 mg/dL). However, she was observed with administration of cefepime. After another 3 days, she developed shock and the abdomen appeared distended. Vital signs were: temperature, 38.1 °C; heart rate, 140 beats/min; blood pressure, 60/40 mmHg under medication with dopamine 10 μg/kg/min and noradrenaline 0.15 μg/kg/min; and oxygen saturation, 92 % in room air. Laboratory data showed: white cell count, 18,100/μL; platelet count, 29,000/μL; CRP, 4.1 mg/dL. CT showed massive ascites, but no free air at that time (Fig. 5). Aspirated ascites showed intestinal juice, so emergency surgery was performed under a diagnosis of gastrointestinal perforation.

**Fig. 6:**
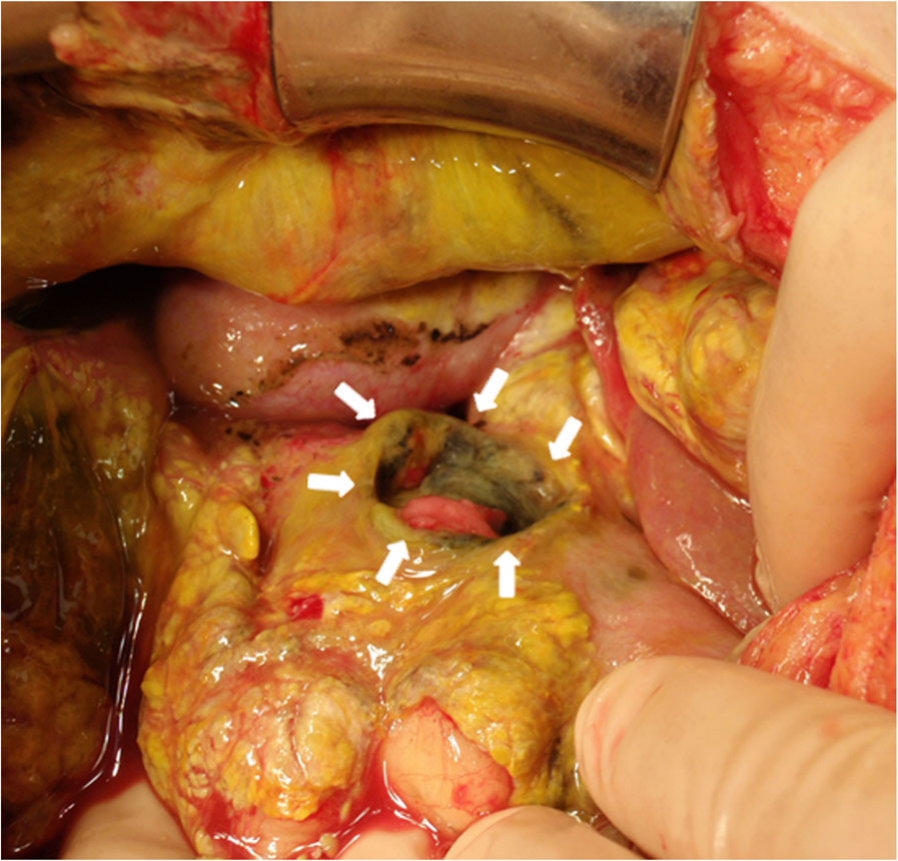
The intra-operative finding. An 4-cm perforation was noted at the anterior wall of the duodenval bulb

On laparotomy, 4 L of biliary ascites was removed, and a perforation 4 cm in length was found at the duodenal bulb (Fig. 6). An omental patch over the perforation site, insertion of a drainage tube into the duodenum from the anterior wall of the stomach, and cholecystectomy with insertion of a bile drainage tube from the cystic duct were performed. Fifteen days after the ulcer operation, continuous bleeding at the wall edge of the duodenal ulcer required reoperation. Operative findings revealed ulcer bleeding and dehiscence of the perforation site. Gastric disconnection was performed, comprising antrectomy including resection of the ulcerated portion, tube duodenostomy, and tube gastrostomy. The patient also needed intensive care, and her condition improved after 3 months. However, digestive reconstruction surgery has not yet been performed as of the time of writing, as the brain tumor recurred during recovery.

## Discussion

In these two cases, the brainstem tumors might have been related to duodenal ulcer perforation that progressed to septicemia. In 1841, Rokitansky suggested for the first time that ulcerative processes of the stomach might involve dysfunction of nervous mechanisms [3]. In 1932, Cushing reported gastroduodenal ulcers produced by elevated intracranial pressure caused by an intracranial tumor, head injury, or other space-occupying lesion. He suggested that such ulcerative processes might be related to diencephalic or brainstem disorders affecting the parasympathetic nervous system. Since then, ulcers of this type have been called Cushing’s ulcers [1].

The mechanism of ulceration appears to involve three routes from the central nervous system to the stomach: 1) anterior hypothalamus – vagus nerve; 2) posterior hypothalamus – sympathetic nerve; and 3) posterior hypothalamus – anterior pituitary gland – adrenal cortex. Through these three routes, factors that aggravate the stomach are increased or protective factors are decreased. The sympathetic and parasympathetic nervous systems usually maintain a balance of the blood supply, gastric secretion, and gastric motility. Dysfunction of the central nervous system stimulates the hypothalamus, which then stimulates the sympathetic and parasympathetic nervous systems. Stimulation of sympathetic nerves decreases blood supply to the stomach, and stimulation of parasympathetic nerves increases gastric secretion. Moreover, adrenal cortical hormones through the anterior pituitary gland decrease gastric mucus secretion [4–10]. These factors then contribute to the development of gastroduodenal ulcers.

In general, factors such as advanced age, concomitant disease, preoperative shock, large size of the perforation, and delays in presentation and operation have been identified as risk factors for mortality in duodenal ulcer perforation [2]. Based on these factors, several scoring systems have been used to evaluate the condition of the patient with duodenal ulcer perforation, such as the Boey score [11], Mannheim Peritonitis Index [12–14], APACHE II score [15] and Jabalpur score [16]. In particular, the perforation-operation interval seems to represent an important factor for mortality. Mishra et al. reported that the mortality rate is 3 % within 24 h, 57 % from 25 to 72 h, and 80 % over 120 h after duodenal ulcer perforation [16]. Many reports have stated that an interval to operation larger than 24 h increases the mortality rate [17–19], because heavier bacterial contamination occurs in patients with delayed treatment [20]. In the present two cases, decreased level of consciousness was the major cause of delayed diagnosis in both patients. Although gauging the interval since ulcer perforation is difficult, at least 48 h may have elapsed in both cases, given the presence of septic shock. Of the above risk factors, our two cases showed large perforations, delayed diagnosis, concomitant disease, and preoperative shock, as well as advanced age in Case 2. Operations in such cases are generally difficult. Nonetheless, antrectomy with Billroth II reconstruction was performed for Case 1 and omental patching was performed for Case 2. Because gastric disconnection requires a second operation for digestive reconstruction, we hesitated to perform this procedure, but gastric disconnection was unavoidable at the first emergency surgery.

Most duodenal ulcer perforations are less than 1 cm in length, and can be successfully treated with one-layer closure plus a pedicled omental patch (Cellan-Jones technique) or an omental patch repair (Graham technique) [21–23]. On the other hand, giant duodenal ulcers are uncommon, with duodenal ulcer perforation more than 3 cm in length reportedly accounting for about 1.23 % of cases [2]. Giant duodenal ulcers are difficult to manage and are associated with high rates of both morbidity (20–70 %) and mortality (15–40 %) because of the extensive duodenal tissue loss and surrounding tissue inflammation [24]. The Cellan-Jones and Graham techniques often fail to achieve closure of the perforation, resulting in postoperative leakage or gastric outlet obstruction.

Several reports have described surgical procedures for giant ulcers, including partial gastrectomy, jejunal serosal patch [25], free omental plug [26], and jejunal pedicle graft [27]. Lal et al. reported the efficacy of triple-tube-ostomy (tube gastrostomy, retrograde tube duodenostomy, and feeding jejunostomy) with repair of the perforation for large duodenal ulcer perforations [28]. Cranford et al. advocated gastric disconnection with truncal vagotomy, antrectomy, and triple-tube-ostomy [29]. This surgical approach is considered the most appropriate procedure for giant duodenal ulcer perforation in cases with poor general conditions owing to late diagnosis. Because one of the present cases showed bleeding and leakage from the repaired duodenal ulcer, antrectomy including the ulcerative portion was thought to be necessary for giant duodenal ulcer. In cases with poor general conditions owing to late diagnosis, digestive tract reconstruction is hazardous, and gastric disconnection might be needed. This approach necessitates a second elective operation for digestive reconstruction, but is thought to represent the safest procedure given the high mortality rate of this condition. Moreover, cholecystectomy with insertion of a bile drainage tube from the cystic duct might also be necessary in preparation for duodenal stump leakage.

## Conclusion

We have reported two cases of giant duodenal ulcer perforation after neurosurgery that needed reoperations because of postoperative leakage and bleeding. For giant duodenal ulcer with poor general condition owing to late diagnosis, simple closure including omental patching or antrectomy with reconstruction is hazardous. Antrectomy with gastric disconnection, which means gastrostomy, duodenostomy, feeding jejunostomy and cholecystectomy, is recommended.

## Abbreviations

CRP: C-reactive protein
CT: Computed tomography
